# Reporting and Representation of Participant Race and Ethnicity in National Institutes of Health–Funded Pediatric Clinical Trials

**DOI:** 10.1001/jamanetworkopen.2023.31316

**Published:** 2023-08-30

**Authors:** Lois K. Lee, Claire Narang, Chris A. Rees, Ravi R. Thiagarajan, Patrice Melvin, Valerie Ward, Florence T. Bourgeois

**Affiliations:** 1 Division of Emergency Medicine, Boston Children’s Hospital, Boston, Massachusetts; 2 Department of Pediatrics, Harvard Medical School, Boston, Massachusetts; 3 Sandra L. Fenwick Institute for Pediatric Health Equity and Inclusion, Boston, Massachusetts; 4 Office of Health Equity and Inclusion, Boston Children’s Hospital, Boston, Massachusetts; 5 Pediatric Therapeutics and Regulatory Science Initiative, Computational Health Informatics Program, Boston Children’s Hospital, Boston, Massachusetts; 6 Division of Pediatric Emergency Medicine, Emory University School of Medicine, Children’s Healthcare of Atlanta, Atlanta, Georgia; 7 Department of Cardiology, Boston Children’s Hospital, Boston, Massachusetts; 8 Department of Radiology, Boston Children’s Hospital, Boston, Massachusetts; 9 Department of Radiology, Harvard Medical School, Boston, Massachusetts

## Abstract

**Importance:**

Enrolling racially and ethnically diverse pediatric research participants is critical to ensuring equitable access to health advances and generalizability of research findings.

**Objectives:**

To examine the reporting of race and ethnicity for National Institutes of Health (NIH)–funded pediatric clinical trials and to assess the representation of pediatric participants from different racial and ethnic groups compared with distributions in the US population.

**Design, Setting, and Participants:**

This cross-sectional study included NIH-funded pediatric (ages 0-17 years) trials with grant funding completed between January 1, 2017, and December 31, 2019, and trial results reported as of June 30, 2022.

**Exposures:**

National Institutes of Health policies and guidance statements on the reporting of race and ethnicity of participants in NIH-funded clinical trials.

**Main Outcomes and Measures:**

The main outcome was reporting of participant race and ethnicity for NIH-funded pediatric clinical trials in publications and ClinicalTrials.gov.

**Results:**

There were 363 NIH-funded pediatric trials included in the analysis. Reporting of race and ethnicity data was similar in publications and ClinicalTrials.gov, with 90.3% (167 of 185) of publications and 93.9% (77 of 82) of ClinicalTrial.gov reports providing data on race and/or ethnicity. Among the 160 publications reporting race, there were 43 different race classifications, with only 3 publications (1.9%) using the NIH-required categories. By contrast, in ClinicalTrials.gov, 61 reports (79.2%) provided participant race and ethnicity using the NIH-specified categories (*P* < .001). There was racially and ethnically diverse enrollment of pediatric participants, with overrepresentation of racial and ethnic minority groups compared with the US population.

**Conclusions and Relevance:**

This cross-sectional study of NIH-funded pediatric clinical trials found high rates of reporting of participant race and ethnicity, with diverse representation of trial participants. These findings suggest that the NIH is meeting its directive of ensuring diverse participant enrollment in the research it supports.

## Key Points

**Question:**

To what extent are participant race and ethnicity reported in publications and ClinicalTrials.gov, and how representative of the US population are participants in National Institutes of Health (NIH)–funded pediatric clinical trials?

**Findings:**

In this cross-sectional study of 363 NIH-funded pediatric clinical trials, reporting of race and ethnicity was high in both publications and ClinicalTrials.gov. Enrollment of pediatric trial participants was racially and ethnically diverse, with overrepresentation of racial and ethnic minority groups compared with the US population.

**Meaning:**

The findings suggest that NIH policies have been effective in ensuring reporting of participant race and ethnicity in trial publications and reports, with NIH-funded pediatric trials enrolling diverse participants representative of the US population.

## Teaser

This cross-sectional study assesses reporting of race and ethnicity in National Institutes of Health (NIH)–funded pediatric clinical trials in publications and ClinicalTrials.gov.

## Introduction

Enrollment of sociodemographically diverse patient populations in clinical trials is a national priority in the US to ensure generalizability of research findings and equitable access to medical advances by all patient populations.^1^ Differences in therapeutic responses between patient populations related to genetic, environmental, and cultural factors have been well documented across drug types and conditions.^2^ Furthermore, trust in clinical research may be increased among patients and their caregivers when clinical trial findings are based on representative patient populations^1^ and the effectiveness of treatments are understood in specific populations.^3^ When possible, the sociodemographic characteristics of patients involved in studies should mirror the populations affected by the disease and intended to receive the intervention under study. This is within the important limitation of understanding that race is a social, not biological, construct.^4,5^ However, race and ethnicity are 1 type of available measure of participant demographics for which the National Institutes of Health (NIH) has established guidelines to promote the inclusion of diverse populations in clinical research.^6,7^

In the US, the NIH is the largest source of biomedical research funding and has an outsized influence on advancing the diversity of patient populations in research. Since passage of the NIH Revitalization Act of 1993, which directed the NIH to establish guidelines for the inclusion of women and minority patients in clinical research,^6^ the NIH has had policies and guidance statements in place on the reporting of race and ethnicity for patients in NIH-funded clinical trials. Reporting of race and ethnicity in clinical trials is essential to promote equity and diversity in research, which ultimately contributes to equitable health care delivery. In 2016, the NIH published a final rule for clinical trials registration and results information submission,^8^ which specified submission of demographic characteristics, including race and ethnicity, as part of mandated reporting of clinical trial results in ClinicalTrials.gov. Reporting requirements were further clarified in guidance released by the NIH in 2017 that specified required reporting of valid analyses for trial outcome measures by race and ethnicity,^9^ as defined by the US Office of Management and Budget.^10^

Despite these activities, a 2022 report from the National Academies of Sciences, Engineering, and Medicine on the representation of women and underrepresented groups in clinical research concluded that racial and ethnic diversity in clinical trials remained inadequate and largely unchanged over the past 30 years.^1^ Although the report did not specifically examine pediatric trial participants, several studies have assessed racial and ethnic diversity in pediatric studies across a spectrum of trial cohorts and presented mixed findings on the level of representation of different patient groups.^11,12,13,14,15,16^ Given the unique considerations in enrolling children into clinical trials, a focused assessment of the diversity of pediatric participants in NIH-funded clinical trials, including reporting practices for race and ethnicity information, could inform the impact of current policies and identify any remaining gaps in the NIH’s strategies.

The objectives of this study were to examine the reporting of race and ethnicity in NIH-funded pediatric clinical trials and analyze the representation of pediatric participants from different racial and ethnic groups compared with distributions in the US population. We hypothesized that there would be variability in the reporting of race and ethnicity and underrepresentation of children from marginalized racial and ethnic groups.

## Methods

We conducted a cross-sectional study of pediatric clinical trials funded by the NIH, analyzing trials with grant funding completed from January 1, 2017, to December 31, 2019, and with results reported by June 30, 2022. The study was exempted from review by the Boston Children’s Hospital Institutional Review Board as it was considered not to meet the criteria of human participants research. This study followed the Strengthening the Reporting of Observational Studies in Epidemiology (STROBE) reporting guideline for cross-sectional studies.

### Selection of Pediatric Clinical Trials

Pediatric grants were identified using the NIH Research Portfolio Online Reporting Tools Expenditures and Results (RePORTER), an online database of NIH-funded research grants.^17^ Using methods previously described,^18^ we selected grants designated as funding pediatric research with original completion dates from January 1, 2017, to December 31, 2019. This study period was selected to allow for sufficient time between funding and trial completion and publication. Each grant abstract was manually reviewed to select pediatric clinical trials, defined as trials prospectively enrolling pediatric patients (age <18 years) and assessing the efficacy and/or safety of an intervention on a pediatric health outcome.^19^ From this cohort, we included trials enrolling pediatric patients in at least 1 US trial site.

For each pediatric clinical trial, systematic searches were performed in PubMed by 2 investigators (C.N. and C.A.R.) to identify associated trial publications. If no publication was identified, we emailed principal investigators for trial publication status (n = 213, with response rate of 68%). For each trial, we also reviewed the ClinicalTrials.gov record for any trial results reported in the registry.^20^ ClinicalTrials.gov provides structured results-reporting tables including information on participant race and ethnicity. A final publication search and review of ClinicalTrials.gov records was performed on June 30, 2022.

### Trial Characteristics and Reporting of Participant Race and Ethnicity

The following trial characteristics were extracted from NIH RePORTER, publications, and ClinicalTrials.gov: year of trial start (ie, date that first participant was enrolled), NIH funding institute, condition studied, intervention type, randomization status, sample size, participant age group, and trial end point. If a grant received funding from multiple NIH institutes (n = 11), it was categorized according to the institute providing the greatest funding. Conditions were classified using a modified version of the Institute of Health Metrics and Evaluation level 2 conditions.^21^ Sample size was preferentially extracted from publications, followed by ClinicalTrials.gov and grant abstracts if a publication was not available. Pediatric age groups were neonates (birth to 1 month), infants (1 month to 2 years), children (3-12 years), and adolescents (13-17 years). Trial end points were defined as clinical outcomes, surrogate measures, and clinical scales.^16,22,23^

Participant race and ethnicity data were reviewed and extracted from publications and ClinicalTrials.gov. For each data source, we determined whether race and ethnicity were reported, how race and ethnicity were categorized and reported, and what method had been used to determine participant race and ethnicity in the trial (eg, self-report). In particular, for race, we determined whether the required NIH categorization of American Indian/Alaska Native, Asian, Black, Native Hawaiian/Pacific Islander, and White was used.^24^ For ethnicity, we recorded whether ethnicity was reported using a stand-alone variable for ethnicity, as required by the NIH,^24^ or whether it was reported as part of a race variable. We recorded the number of participants enrolled from each racial and ethnic category using data from publications, followed by ClincialTrials.gov if a publication was not available. In ClinicalTrials.gov, we used the Baseline Characteristics Module in the results section, which provides patient demographic information in structured tables.^9^ When possible, we counted participants separately according to race category and ethnicity. To compare distributions of race and ethnicity among trial participants with the US population, demographic information for individuals aged 0 to 17 years was obtained from US census data estimates through the Kids Count Data Center for 2021.^25^

### Statistical Analyses

We calculated descriptive frequencies for trial characteristics and reporting of race and ethnicity with medians, IQRs, and SDs. We performed comparative statistics with χ^2^ or Fisher exact tests to compare reporting between publications and ClinicalTrials.gov. To compare the race and ethnicity composition of participants in pediatric clinical trials with the US population, we conducted univariate logistic regression, reporting odds ratios (OR) with 95% CIs. These analyses excluded participants with unknown race or race reported as “other” since there are no corresponding categories in the US census data. For studies collecting both race and ethnicity information, participants who were Hispanic or Latino could contribute both race and ethnicity data but were counted only once in the sample size. Two studies with missing sample sizes were excluded from these analyses. In a sensitivity analysis, we examined the race and ethnicity distributions of participants excluding trials targeting enrollment of specific racial or ethnic groups. All analyses were conducted in SAS, version 9.4 (SAS Institute), with 2-sided *P* < .05 considered to be statistically significant.

## Results

There were 363 NIH-funded pediatric clinical trials conducted in the US that met our inclusion criteria. Among these, 208 trials representing 99 652 participants reported clinical trial results in a peer-reviewed publication (n = 185) or ClinicalTrials.gov (n = 82), with 59 reporting in both (Figure). Trials were started in 2011 to 2019 and published between 2016 and 2022, with 151 (82%) published from 2019 to 2022. The Eunice Kennedy Shriver National Institute of Child Health and Human Development was the most frequent funding institute (67 trials [32.2%]) followed by the National Heart, Lung, and Blood Institute (27 [13.0%]) (Table 1). The most frequently studied conditions were mental health disorders (53 trials [25.5%]), obesity (37 [17.8%]), and substance use (23 [11.1%]). There were 154 trials (74.0%) studying a behavioral intervention and 158 (76.0%) that were randomized clinical trials. The median trial sample size was 160 participants (IQR, 62-339 participants). Only 52 trials (25.0%) enrolled individuals younger than 3 years, while 51 (24.5%) enrolled exclusively adolescents aged 13 to 17 years.

**Figure.**
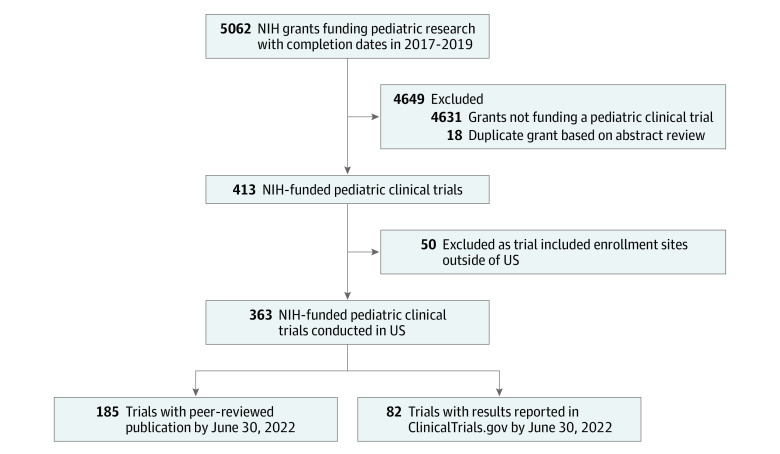
Study Flow Diagram Flow diagram for selection of National Institutes of Health (NIH)–funded pediatric clinical trials with publications or results reported in ClinicalTrials.gov.

**Table 1.** Characteristics of National Institutes of Health–Funded Pediatric Clinical Trials

Characteristic	Clinical trials, No. (%) (N = 208)
Funding institute	
NICHD	67 (32.2)
NHLBI	27 (13.0)
NIMH	27 (13.0)
NIDA	17 (8.2)
NIDDK	14 (6.7)
NCI	12 (5.8)
NIAAA	10 (4.8)
Other	45 (16.3)
Conditions studied	
Mental health disorders	53 (25.5)
Obesity	37 (17.8)
Substance use	23 (11.1)
Neonatal conditions	19 (9.1)
Chronic respiratory diseases	11 (5.3)
Other	65 (31.3)
Intervention type	
Behavioral	154 (74.0)
Drug	29 (13.9)
Device	10 (4.8)
Diagnostic test	6 (2.9)
Procedure	6 (2.9)
Dietary supplement	4 (1.4)
Randomization status	
Randomized	158 (76.0)
Cluster randomized	30 (14.4)
Not randomized	20 (9.6)
Sample size^a^	
<100	74 (35.7)
100-299	72 (34.8)
>300	61 (29.5)
Youngest participant age group	
Birth to 1 mo	36 (17.3)
1 mo to 2 y	16 (7.7)
3-12 y	105 (50.5)
13-17 y	51 (24.5)
Trial end point	
Clinical outcome	86 (41.4)
Surrogate measure	82 (39.4)
Clinical scale	40 (19.2)

Abbreviations: NCI, National Cancer Institute; NHLBI, National Heart, Lung, and Blood Institute; NIAAA, National Institute on Alcohol Abuse and Alcoholism; NICHD, Eunice Kennedy Shriver National Institute of Child Health and Human Development; NIDA, National Institute on Drug Abuse; NIDDK, National Institute of Diabetes and Digestive and Kidney Diseases; NIMH, National Institute of Mental Health.

^a^ N = 207 due to 1 trial with missing sample size.

### Race and Ethnicity Reporting in Publications and ClinicalTrials.gov

Among 185 clinical trial publications, 167 (90.3%) included information on race and/or ethnicity: 160 (86.5%) on participant race and 131 (70.8%) on ethnicity (Table 2). The proportion of trials reporting race and ethnicity was higher in ClinicalTrials.gov compared with publications, with 77 of 82 trial reports (93.9%) providing data on race (*P* = .08 vs publications) and 64 (78.0%) providing data on ethnicity (*P* = .22 vs publications). For publications, race categories used for reporting varied widely, with a total of 43 different classifications. The most common reporting format consisted of Black, White, or other (35 trials [21.9%]) followed by White only (12 [7.5%]). Only 3 publications (1.9%) presented race categories using the 5 NIH-required categories. By contrast, in ClinicalTrials.gov, 61 trial reports (79.2%) included participant race using the NIH-specified categories (*P* < .001 vs publications). Accordingly, the reporting of data for each of the racial minority categories was significantly higher in ClinicalTrials.gov compared with publications (Table 2).

**Table 2.** Reporting of Race and Ethnicity Information in Publications and ClinicalTrials.gov

	Publications (n = 185)	ClinicalTrials.gov (n = 82)	*P* value
Data reported			
Any race	160 (86.5)	77 (93.9)	.08
Any ethnicity	131 (70.8)	64 (78.0)	.22
Either race or ethnicity	167 (90.3)	77 (93.9)	.33
Race categories used for reporting^a^			
American Indian/Alaska Native, Asian, Black, Native Hawaiian/Pacific Islander, White, multiracial, or unknown^b^	3 (1.9)	61 (79.2)	<.001
American Indian/Alaska Native, Asian, Black, White, or other	5 (3.1)	1 (1.3)	.67
Asian, Black, White, or multiracial	10 (6.3)	0	.03
Asian, Black, White, or other	10 (6.3)	1 (1.3)	.11
Black, White, or multiracial	7 (4.4)	0	.10
Black, White, or other	35 (21.9)	1 (1.3)	<.001
Black, White, other, or multiracial	10 (6.3)	1 (1.3)	.11
Black only	10 (6.3)	0	.03
White only	12 (7.5)	0	.01
Other race categories^c^	58 (36.3)	12 (15.6)	.001
Reporting of data for individual race categories^a^			
American Indian/Alaska Native	38 (23.8)	69 (89.6)	<.001
Asian	55 (34.4)	70 (90.9)	<.001
Black	137 (85.6)	75 (97.4)	.01
Native Hawaiian/Pacific Islander	18 (11.3)	65 (84.4)	<.001
White	144 (90.0)	76 (98.7)	.01
Hispanic or Latino ethnicity category used for reporting^d^	131 (100)	64 (100)	NA
Reporting format for ethnicity^d^			
Reported as ethnicity	81 (61.8)	58 (90.6)	<.001
Reported as race	41 (31.3)	6 (9.4)	
Unclear	9 (6.9)	0	
Source of race and ethnicity data^e^			
Parent report	59 (35.3)	0	<.001
Self-report	28 (16.8)	0	
Self- or parent report	5 (3.0)	0	
School report	2 (1.2)	0	
Not specified	73 (43.7)	77 (100)	

Abbreviation: NA, not applicable.

^a^ Limited to 160 publications and 77 ClinicalTrials.gov reports with race data.

^b^ National Institutes of Health standard.

^c^ Represents 34 additional categorizations for reporting race.

^d^ Limited to 131 publications and 64 ClinicalTrials.gov reports with ethnicity data.

^e^ Limited to 167 publications and 77 ClinicalTrials.gov reports with either race or ethnicity data.

Information on participant ethnicity was reported as Hispanic or Latino for all trials. Ethnicity was reported as a separate ethnicity variable (as opposed to a category within a race variable) in 81 publications (61.8%) and in 58 ClinicalTrials.gov reports (90.6%) (*P* < .001). It was reported as a category within a race variable in 41 publications (31.3%), but only in 6 ClinicalTrials.gov reports (9.4%). The source of race and ethnicity data was available in 94 publications (50.8%) and was not specified in any ClinicalTrials.gov reports. Among publications, the source was parent report in 59 (35.3%) and self-report in 28 (16.8%); however, it was not specified in 73 (43.7%).

### Racial and Ethnic Composition of Participants in Pediatric Clinical Trials

Participants in clinical trials were well represented across race and ethnicity groups compared with race and ethnicity distributions in the US population (Table 3). The proportion of White pediatric patients (43.0%) enrolled in clinical trials was lower than the proportion in the US population (49.6%) (OR, 0.77; 95% CI, 0.76-0.78). There was overrepresentation of American Indian/Alaska Native, Asian, Black, and Native Hawaiian/Pacific Islander participants as well as Hispanic and Latino participants in the clinical trials compared with the US population. Results were similar in sensitivity analyses excluding trials with targeted enrollment of specific racial and ethnic groups, with the exception that American Indian/Alaska Native trial participants were no longer significantly overrepresented (0.8% vs 0.8%; OR, 0.96; 95% CI, 0.89-1.03) (eTable in Supplement 1).

**Table 3.** Racial and Ethnic Composition of Pediatric Clinical Trial Participants^a^

Race or ethnicity	Trial participants (N = 99 652), No. (%)^b^	US census population ( = 72 822 113), No. (%)	Odds ratio (95% CI)
American Indian/Alaska Native	2116 (2.1)	594 670 (0.8)	2.64 (2.52-2.75)
Asian	9257 (9.2)	3 938 157 (5.4)	1.79 (1.75-1.83)
Black	18 403 (18.5)	10 007 204 (13.7)	1.42 (1.40-1.44)
Hispanic or Latino	29 879 (30.0)	18 631 835 (25.6)	1.25 (1.23-1.26)
Native Hawaiian/Pacific Islander	1682 (1.7)	155 618 (0.2)	8.07 (7.64-8.41)
White	42 834 (43.0)	36 133 127 (49.6)	0.77 (0.76-0.78)
Multiracial	4050 (4.1)	3 361 502 (4.6)	0.88 (0.85-0.90)

^a^ Participants with unknown or “other” race were excluded from comparisons as the US census does not include corresponding categories.

^b^ Percentages sum to greater than 100 because certain studies collected information on both race and ethnicity, and for these studies, Hispanic or Latino participants contributed both race and ethnicity data.

## Discussion

In this study examining reporting of participant race and ethnicity in NIH-funded pediatric clinical trials, we found that participant race and ethnicity were reported in the majority of trial publications and in ClinicalTrials.gov. Information on race was more consistently reported in ClinicalTrials.gov, with publications using a wide range of classification schemes and few using the required NIH categorization. Overall, the reporting of information on individual race categories was greater for all underrepresented groups in ClinicalTrials.gov compared with publications. Ethnicity was universally reported as Hispanic or Latino, although 31.3% of publications reported ethnicity as part of a racial demographic. In contrast, in ClinicalTrials.gov, 90.6% of trials provided data using a stand-alone ethnicity variable, as required by the NIH. Trials demonstrated racially and ethnically diverse participant enrollment, with overrepresentation of American Indian/Alaska Native, Asian, Black, and Native Hawaiian/Pacific Islander participants as well as Hispanic and Latino participants compared with the US population.

The NIH has specified race and ethnicity categories for the collection and reporting of participant race and ethnicity in clinical trials.^26^ However, we found that 43 categorization schemes were used for race alone among publications, while ethnicity was not consistently reported as a separate demographic characteristic. This variability is likely the result of a number of factors, including the prevalence of studied conditions in different populations, demographic characteristics of patients in specific geographic locations, and lack of standardized race and ethnicity categorization in journals. Patient databases across health care systems also do not use a single system for collecting race and ethnicity, nor do other federal health care agencies or private entities, such as health care insurance companies.^27^ This lack of consistent reporting across health care databases may be contributing to the variation in reporting by investigators in journal publications. A potential concern with the current approach is that race and ethnicity reporting in publications may be incomplete or may not be comparable across studies.

In ClinicalTrials.gov, adherence to NIH reporting requirements was higher for both race and ethnicity compared with publications. The NIH has published detailed guidelines to support investigators in reporting results in this registry and in providing complete and consistent results for stratified populations.^9^ Our analysis suggests that these efforts have been largely successful, with race and ethnicity reported according to NIH specification for 79.2% and 90.6% of trials, respectively. The lack of adherence among certain trials may be related to the manner in which the data were originally collected. The NIH should continue to monitor the quality of participant race and ethnicity reporting to ensure that ongoing adoption of its guidelines will reduce these remaining discrepancies. In addition, to appropriately capture the complex demographics of patient populations in the US, best practices are needed for the collection and reporting of these data.^28,29^ The US Office of Budget and Management announced in January 2023 that they will be undertaking this work, with the launch of a new proposal for the collection and presentation of race and ethnicity data to more accurately reflect the diverse population in the US.^30^

The NIH recommends patient self-report as the preferred approach to collect race and ethnicity information,^24^ although specific guidance on exact methods for collecting this information is limited.^31,32^ Data collected by observers are at high risk for inaccuracy,^33,34^ although self-reported data may also be prone to misclassification if standard race and ethnicity categories are not used.^34^ Collecting this information from children’s guardians using standardized categories has been proposed as the best approach to determine pediatric race and ethnicity.^12,35^ In this study, among publications, race and ethnicity data were described as collected by a parent in 35.3% of studies, although the source was not specified for 43.7% of trials. ClinicalTrials.gov does not include a data field for investigators to indicate how this demographic information was collected, and addition of this variable could be considered in future iterations of the trial registry.

The diversity of children included in clinical research has been examined in prior studies with varying results regarding representation of different racial and ethnic groups.^11,12,16,36,37^ One study analyzing data from 33 federally funded studies evaluating pediatric drugs and devices and enrolling almost 11 000 participants from 2008 to 2020 found no evidence of racial or ethnic bias in enrollment.^12^ Another study analyzed 612 pediatric clinical trial results in leading medical journals from 2011 to 2020 and found that while there was overrepresentation of Black and Hispanic children compared with the US population, American Indian/Alaska Native, Asian, and Native Hawaiian/Pacific Islander children were underrepresented.^16^ Our analysis, limited to NIH-funded pediatric clinical trials and capturing a more recent publication period, showed overrepresentation of racial and ethnic minority groups compared with the US population. This shift may reflect the effectiveness of NIH policies and activities by other medical and research organizations as well as increasing awareness among investigators on the importance of recruiting racially and ethnically diverse clinical trial participants.

It is important to note that we examined only race and ethnicity as opposed to other sociodemographic features, as race and ethnicity are most consistently available in clinical trial reports and subject to specific NIH reporting requirements. As a social construct, race categorizes individuals based on physical characteristics, and it is different from and should not be considered a proxy for geographic, biologic, or genetic ancestry.^4,5,38,39,40^ Thus, using these categories alone is insufficient to explain differences in clinical trial outcomes. Structural and social determinants, including socioeconomic, environmental, and societal factors, along with individual lived experiences, must also be considered.^4,5,38,39,40,41^ Moving forward, clinical research should advance the understanding of the influences of genetic and biologic characteristics as well as of the experiences of racism and of social determinants of health^4^ by including socioeconomic context measures, such as the Social Vulnerability Index^42^ or the Childhood Opportunity Index.^43^

### Limitations

This study has several limitations. Our analysis was limited to pediatric trials funded by the NIH, and results may not be generalizable to studies funded through other sources or not focused on pediatric populations. In comparing race and ethnicity distributions between trial participants and the US population, participants with unknown or “other” race could not be included in the analysis as the US census does not have corresponding categories. Furthermore, we were not able to account for the racial and ethnic distribution in a study catchment area, which may have differed from the national average. Finally, we relied exclusively on publicly reported data in publications and ClinicalTrials.gov and were unable to ascertain the accuracy of the data reported.

## Conclusions

In this cross-sectional study, the majority of NIH-funded pediatric clinical trials reported race and ethnicity information in trial publications and ClinicalTrials.gov. While there was high adherence to the use of NIH-required race and ethnicity categories among trial results reported in ClinicalTrials.gov, we found substantial variation in reporting formats used in publications. Overall, the racial and ethnic diversity of pediatric participants in clinical trials suggests that the NIH is meeting its directive of ensuring diverse participant enrollment in the research it supports. Future initiatives could focus on increasing the use of standard categories in medical journals to ensure complete and consistent reporting of race and ethnicity in trial publications.

## Notes

Supplement 1. **eTable.** Racial and Ethnic Composition of Pediatric Clinical Trial Participants, Excluding Trials Targeting Specific Races and/or Ethnicities

Supplement 2.
Data Sharing Statement
