# Supplementary material for: Reporting and Representation of Participant Race and Ethnicity in National Institutes of Health–Funded Pediatric Clinical Trials
Source: JAMA Netw Open. 2023 Aug 30;6(8):e2331316. doi: 10.1001/jamanetworkopen.2023.31316 (PMC10469249; doi:10.1001/jamanetworkopen.2023.31316)
Supplement: Supplement 1. — eTable. Racial and Ethnic Composition of Pediatric Clinical Trial Participants, Excluding Trials Targeting Specific Races and/or Ethnicities [file jamanetwopen-e2331316-s001.pdf]

Supplemental Online Content

Lee LK, Narang C, Rees CA, et al. Reporting and representation of participant race and ethnicity in National Institutes of Health–funded pediatric clinical trials. *JAMA Netw Open.* 2023;6(8):e2331316. doi:10.1001/jamanetworkopen.2023.31316

eTable. Racial and Ethnic Composition of Pediatric Clinical Trial Participants, Excluding Trials Targeting Specific Races and/or Ethnicities

This supplemental material has been provided by the authors to give readers additional information about their work.

© 2023 Lee LK et al. *JAMA Network Open.*

eTable. Racial and Ethnic Composition of Pediatric Clinical Trial Participants, Excluding Trials Targeting Specific Races and/or Ethnicities[1]

Race and ethnicity[2]	Trial participants (N=94 789), No. (%)[3]	US Census Population (=72 822 113), No. (%)	Odds ratio (95% CI)
American Indian/Alaska Native	744 (0.8)	594 670 (0.8)	0.96 (0.89-1.03)
Asian	8642 (9.1)	3 938 157 (5.4)	1.75 (1.72-1.79)
Black	16 954 (17.9)	10 007 204 (13.7)	1.37 (1.34-1.39)
Hispanic/Latino	28 139 (29.7)[3]	18 631 835 (25.6)	1.23 (1.21-1.25)
Native Hawaiian/Pacific Islander	1362 (1.4)	155 618 (0.2)	6.81 (6.45-7.18)
White	42 123 (44.4)	36 133 127 (49.6)	0.81 (0.80-0.82)
Multiracial	4045 (4.3)	3 361 502 (4.6)	0.92 (0.89-0.95)

[1] There were 186 trials included in the analysis after excluding 6 trials targeting enrollment of Black children, 7 of American Indian/ Alaska Native children, and 9 of Hispanic/Latino children.

[2] Participants with unknown or “other” race were excluded from comparisons as the US Census does not include corresponding categories.

[3] Percentages sum to greater than 100 because certain studies collected information on both race and ethnicity, and for these studies Hispanic/Latino participants contributed both race and ethnicity data.

© 2023 Lee LK et al. *JAMA Network Open.*
