# Supplementary material for: Reporting and Representation of Participant Race and Ethnicity in National Institutes of Health–Funded Pediatric Clinical Trials
Source: JAMA Netw Open. 2023 Aug 30;6(8):e2331316. doi: 10.1001/jamanetworkopen.2023.31316 (PMC10469249; doi:10.1001/jamanetworkopen.2023.31316)
Supplement: Supplement 2. — Data Sharing Statement [file jamanetwopen-e2331316-s002.pdf]

Data Sharing Statement

Lee. Reporting and Representation of Participant Race and Ethnicity in National Institutes of Health–Funded Pediatric Clinical Trials. *JAMA Netw Open*. Published August 30, 2023. doi:10.1001/jamanetworkopen.2023.31316

Data
Data available: Yes
Data types: Data (not involving human participants)
How to access data: All data analyzed in this study are publicly available in publications and in ClinicalTrials.gov. Investigators seeking additional information may contact the authors.
When available: With publication

Supporting Documents
Document types: None

Additional Information
Who can access the data: Anyone
Types of analyses: Data can be used for any purpose
Mechanisms of data availability: Online access
